# Prognostic value of Tpeak-Tend interval in patients with acute pulmonary embolism

**DOI:** 10.1186/s12872-015-0091-4

**Published:** 2015-09-03

**Authors:** Abdullah Icli, Mehmet Kayrak, Hakan Akilli, Alpay Aribas, Mukremin Coskun, Sumeyye Fatma Ozer, Kurtulus Ozdemir

**Affiliations:** Department of Cardiology, Necmettin Erbakan University Meram School of Medicine, Meram, Konya Turkey

## Abstract

**Background:**

The aim of this study was to examine the Tpeak-Tend (Tpe/corrected Tpe) interval, which is an indicator of transmural myocardial repolarization, measured non-invasively via electrocardiogram in patients with acute pulmonary embolism (PE), and to investigate the relationship with 30-day mortality and morbidity.

**Methods:**

The study included 272 patients diagnosed with acute PE, comprising 154 females and 118 males, with a mean age of 63.1 ± 16.8 years. Tpe/cTpe intervals were calculated from the electrocardiograms with a computer program after using a ruler or vernier caliper manual measuring tool to obtain highly sensitive measurements. The relationship between the electrocardiogram values and 30-days mortality and morbidity were measured.

**Results:**

The study group was divided into three groups according to cTpe intervals: Group 1, < 113 ms; Group 2, 113–133 ms; and Group 3, > 133 ms. White blood cell count and troponin T levels, corrected QT intervals with QRS complex durations, percentage of right ventricle dilatation with right/left-ventricular ratio, 30-day death, and combinations of these values were seen at a higher rate in Group 3 patients compared to the other groups. Kaplan–Meier analysis showed that the cTpe interval measured at > 126 ms could be used as a cut-off value in the prediction of mortality and morbidity. The cTpe cut-off values of 126 ms had sensivity, specificity, negative predictive value, and positive predictive value of 80.56 %, 59.32 %, 95.2 %, and 23.2 %, respectively.

**Conclusions:**

cTpe interval could be a useful method in early risk stratification in patients with acute PE.

## Background

Despite the development of several diagnostic and treatment methods for acute pulmonary embolism acute (PE), the condition continues to be a significant cause of cardiovascular morbidity and mortality [1, 2]. According to current guideline recommendations, risk stratification of patients with acute PE is mandatory to allow assessment of the individual prognosis and to guide therapeutic decision making. As a result, various combinations of clinical findings with imaging and laboratory tests have been proposed and tested in registries and cohort studies in an attempt to improve risk stratification [3, 4]. Electrocardiogram (ECG) is used less in acute PE diagnosis due to the existence of newer diagnostic modalities, such as echocardiography, computed tomography, and angiography [5–7]. The value of ECG has not declined, however, as it is easily conducted, non-invasive, and inexpensive.

ECG used in the setting of acute PE patients may present a wide variety of manifestations. Several ECG markers of ventricular repolarization have been reported to identify high-risk patients with acute PE [8]. The mechanism for the appearance of ventricular repolarization following the onset of acute PE is unknown, but may involve the development of acute cor pulmonale caused by the enlargement of the right ventricle (RV) due to rapid RV pressure overload [9, 10]. In acute PE patients, a sudden increase in RV arterial load leads to reduced cardiac output by impairing left ventricle (LV) circulation, and can lead to evident impairment of coronary perfusion and RV damage [11, 12]. The increase in RV pressure and volume leads to greater wall tension and myocyte stretch; RV contraction time is prolonged, while neurohumoral activation leads to inotropic and chronotropic stimulation [13]. In addition, severe RV ischemia may alter the pathway of electrical repolarization, resulting in inverted T waves [14]. acute PE affects both the circulation and gas exchange. The primary cause of death in severe PE is the result of right ventricular failure due to pressure overload. The prolongation of RV contraction time into early diastole in the LV leads to leftward deviation of the interventricular septum. As a result, LV filling is hindered in early diastole, which may lead to a reduction of the cardiac output and contribute to systemic hypotension and hemodynamic instability. In addition, excessive neurohumoral activation in acute PE can be the result both of abnormal RV wall tension and of circulatory shock [15]. Therefore, the differences in the repolarization of ventricular myocardial consist of course of time (just as in various cardiac diseases, such as the Brugada, short QT, and long QT syndromes) and catecholaminergic polymorphic ventricular tachycardia. Differences in the time-course of the repolarization of these three predominant ventricular myocardial cell types contribute significantly to T-wave morphology. These differences in action potential morphology lead to the development of opposing voltage gradients on either side of the subendocardial (M cell) region, which contributes to the inscription of the T wave: especially those that are inscribed in the precordial lead V5 [16]. Measuring the interval between the peak and the end of the T wave (Tpe) as an index of transmural dispersion of repolarization is a parameter that is thought to be capable of reflecting the dispersion of repolarization, and thus may be used as a prognostic tool for the detection of arrhythmic risk [17].

In this study, we discuss the potential value of Tpe as an index of transmural dispersion of repolarization in patients with acute PE. Prolonged Tpe has been associated with increased risk of mortality in the congenital and acquired long QT syndromes [18] and hypertrophic cardiomyopathy [19], as well as in patients who are undergoing primary percutaneous coronary intervention for myocardial infarction [20]. Information is lacking, however, about assessing Tpe in patients with acute PE. The aim of this study was thus to investigate the predictive value of Tpe/cTpe, measured via ECG in patients with acute PE, for 30-day morbidity and mortality.

## Methods

### Study population

This retrospective study included adult patients who had been diagnosed with acute PE who were treated at our university hospital between January 2010 and June 2014. Of the 340 patients, 300 were confirmed as having PE by pulmonary computed tomography, 22 by ventilation/perfusion scintigraphy, and 18 by transthoracic echocardiography. The study was approved by Ethics Committee of Necmettin Erbakan University Clinical Investigations (date/approval number: 2015.01.16/104). The study was conducted in accordance with the ethical principles described by the Declaration of Helsinki.

### Exclusion criteria

**Fig. 1:**
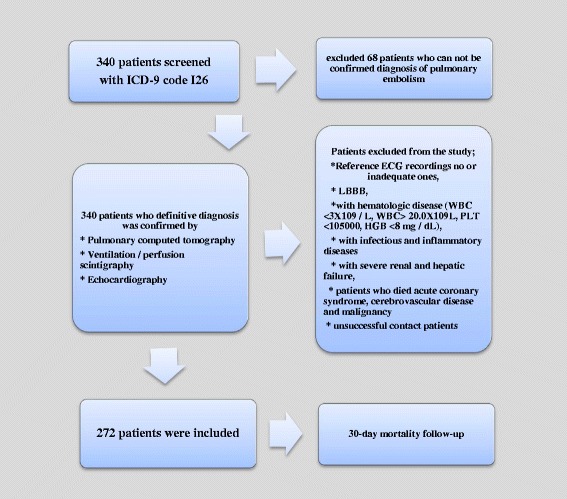
Flow chart of the study design. WBC:white blood cell count; PLT: platelet count; HGB: haemoglobin

Patients were excluded if their ECG was unsuitable for analysis, or if the admission records indicated left bundle branch block or had incomplete echocardiograhy data. Patients with hematological disorders (white blood cell < 3.0–10^9^/L or > 20.0–109/L), infectious or inflammatory diseases, or serious renal or liver disease, as well as those currently using immunosuppressant drugs (including steroids), were excluded from the study. After the exclusion of 68 patients who had PE, 272 patients were found to be eligible for the study (Fig. 1).

### Study protocol

Acute PE cases were screened by ICD-9 code I26 from the hospital’s electronic database. After the initial screening process, the PE diagnostic methods of computed tomography, scintigraphy, or echocardiography were determined, and patients were classified into “definitive acute PE” or “suspicion of acute PE” groups. Suspicious cases were excluded from the analysis. The exclusion criteria were examined in the definitive acute PE group (Fig. 1). Patients who could not be contacted following discharge were excluded. All patients (or their respective relatives) were contacted via telephone and were questioned about 30-day mortality. The study included 272 patients with interpretable ECG recordings, and for whom the prognostic follow-up could be applied for 30 days. The patients comprised 154 females and 118 males, with a mean age of 63.1 ± 16.8 years. Of the 68 patients excluded from the study, 26 had incomplete echocardiography, 8 did not undergo echocardiography, 8 had left brach bundle block in the ECG, 20 had excessive “noise” in the ECG, and 10 had missing laboratory data (Fig. 1).

The digital patient record system was examined in detail for all patients, and a record was made of baseline characteristics (sex, age, etc.), comorbidities, symptoms, hemodynamic conditions, 30-day mortality rates during hospitalization, total hospitalization period, radiographic test results, and laboratory findings. The ECGs and echocardiography taken upon admission were evaluated (Table 1). Geneva and Wells scores were retrospectively assessed for all patients.

**Table 1:** Demographic, clinical and laboratory characteristics of all groups

Variables	Group 1, (n = 87)	Group 2, (n = 93)	Group 3, (n = 92)	*P*
*Demographic features*				
Age.years	58.7 ± 17.7*^a.b.c^	63.8 ± 16.0	66.13 ± 16.00	*0.01*
Gender (Male/Female	45/42	54/39	54/38	0.58
Malignancy. n (%)	14 (16.1)	24 (25.8)	18 (19.6)	0.26
CAD n (%)	9 (10.3)	16 (17.4)	23 (25)	*0.04*
Hypertension. n (%)	26 (30.2)	30 (32.3)	40 (43.5)	0.13
DiabetesMellitus. n (%)	12 (13.8)	16 (17.2)	23 (25)	0.14
*HemodynamicParameters*				
SystolicBp (mmHg)	112.1 ± 18.5	113.0 ± 21.7	109.6 ± 23.7	0.56
DiastolicBp (mmHg)	70.4 ± 10.8	69.9 ± 12.2	70.8 ± 14,0	0.96
Heart Rate (bpm)	87.5 ± 21	107.4 ± 115	118.2 ± 145,2	0.20
O2 saturation (%)	87.3 ± 17.3	87.1 ± 8.5	86.8 ± 15.6	0.98
SHOCK index	0.8 ± 0.2	1,0 ± 0.1	1.1 ± 1.3	0.11
*Laboratory Findings*				
WBC	9.5 ± 3.6	10.7 ± 4.7	12.77 ± 6.3	*0.0001*
Haemoglobin (g/dL)	14.1 ± 15.5	11.7 ± 2.3	12.5 ± 8.9	0.14
TroponinT mg/dL, median (IQR)	0.11(0.17)^a^	0.12(0.24)	0.23(0.48)	*0.03*
*Clinical scores*				
Genevascore	8,8 + 12,5	7,8 + 3,5	7,3 + 3,4	0,44
Wellsscore	5,5 + 2,0	5,4 + 2,0	5,2 + 1,6	0,69
*Echocardiographic measurements*				
*RV dilatation (%)*	27 (50.9)	30 (68.2)	38 (74.5)	*0.04*
EF	59.0 ± 7.5	57.7 ± 7.5	58.6 ± 6.1	0.64
PAB	52.6 ± 21.4	57.1 ± 16.3	54.9 ± 18.3	0.45
RVLV ratio	1,03 ± 0,16	1,16 ± 0,20	1,28 ± 0,23	*0.001*
*clinical outcome for 30 days*				
Death	2 (2.3)	10 (10.8)	24 (26.1)	*0.001*
Combine endpoint	3 (3,5)^*^a	14 (15,1)	25 (27,5)	*0,001*

*^abc ^means that statitically significant differece between subgroups as ^a^: group1 vs. 2, ^b^: group 1 vs 3, ^c^: group 2 vs. 3

^abc ^means that statitically significant differece between subgroups as ^a^: group1 vs. 2, ^b^: group 1 vs 3, ^c^: group 2 vs. 3. 

### Echocardiographic evaluation

Transthoracic echocardiography recordings were obtained from the patients’ records. If a patient’s echocardiography data included all of the required measurements (RV/LV diameters, ejection fraction, or pulmonary artery pressure), the data was defined as complete; otherwise, it was defined as incomplete. In this study, we used the recorded data from comprehensive echocardiograhy performed upon admission on subjects in the left lateral decubitus position. To calculate the RV/LV ratio, archived measurements were used from the mid-cavitary end-diastolic diameters recorded from the standard, apical four-chamber view. In addition, archived data were used of pulmonary artery systolic pressure, which was derived as the sum of the tricuspid regurgitant gradient, obtained from continuous-wave Doppler.

### Definition of adverse clinical events

Our statistical analysis focused on the 30-day period. The adverse clinical events were overall mortality and a complicated course, defined as death or one of the following: the need for catecholamine blood pressure support, cardiopulmonary resuscitation (noninvasive positive pressure ventilation or invasive positive pressure ventilation), or mechanical cardiovascular support due to hemodynamic instability. “Hemodynamic instability” was defined as: (1) PE causing hypotension (systolic blood pressure ≤ 90 mm Hg, or a reduction of at least 40 mm Hg for at least 15 min); (2) tachycardia (pulse rate ≥ 110/min); (3) hypoxemia (PaO2 < 60 mmHg); (4) blurred consciousness; or (5) syncope associated with hypotension, oliguria, or cool extremities. “Shock index” was defined as the ratio of the systolic blood pressure heart rate.

### Measurement of Tpe, cTpe, QT, cQT, and QRS intervals from the 12-Lead ECG

**Fig. 2:**
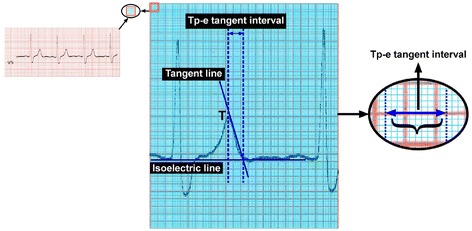
measurement of Tp-e via the tangent method with a computer program

An “appropriate” ECG was defined as one that had at least ten analyzable leads for the required measurements. Otherwise, the ECG was considered to be excessively noisy. ECG measurements were conducted from ECG recorded upon admission using a supine, standard 12-lead ECG tracing at 25 mm/s paper speed at 10 mm/mV amplitude. All ECG recordings were screened through a computer at 800-dpi resolution. The Tpe/cTpe intervals were calculated with a computer program after using a ruler or vernier caliper manual measuring tool to obtain highly sensitive measurements (Fig. 2). Tpe and QT intervals were measured in lead V5. If V5 was not suitable, leads V4 or V6 (in that order) were measured [21]. The end of the T wave was defined as the intersection of the tangent to the down-slope of the T wave and the isoelectric line (when not followed by a U wave or if distinct from the following U wave). If a U wave followed the T wave, the T wave offset was measured as the nadir between the T and U waves. If the T wave amplitude was <1.5 mm in a particular lead, that lead was excluded from the analysis. The QT interval was measured from the earliest onset of the QRS complex (so-named for the Q, R, and S waves in an ECG) to the end of the T wave. In patients with atrial fibrillation and flutter, Tpe was measured in five consecutive beats. All measured intervals were corrected according to the Bazzet formula, which considers heart rate. Patients were divided into three groups based on the tertile of corrected Tpeak-Tend. The lowest tertile included patients whose Tpe/cTpe values ranged from 80–113 ms, the middle tertile ranged from 113–133 ms, and the highest tertile ranged from 134–188 ms.

All ECG measurements were performed by two independent cardiologists who were blinded to other patient information. To estimate intra-observer and inter-observer variability, twenty randomized ECGs were re-analyzed, and the measurements were repeated by two cardiologists. To assess inter-observer variation, all ECG tracings were analyzed by a second, independent investigator who was blinded to the results obtained by the first investigator.

### Statistical analyses

The data were analyzed using SPSS software version 18.0 (SPSS, Chicago, IL, USA) and were presented as mean ± standard deviation. The distribution of the variables was analyzed with the Kolmogorov–Smirnov test. Correlation analysis was carried out with the Spearman correlation test for non-parametrically distributed variables. The differences between the three groups were tested via independent Student’s t-tests for normally distributed variables; the Mann–Whitney U test was used for non-parametrically distributed variables. The differences between the categorical variables were determined by the chi-squared test. The Kaplan–Meier survival analysis was performed to compare the difference of survival between above- and below-median cTpe values, as well as the differences among cTpe tertiles using log-rank tests.

## Results

Of the 68 patients excluded from the study, 26 had incomplete echocardiography, 8 did not undergo echocardiography, 8 had left brach bundle block in the ECG, 20 had excessive noise in the ECG, and 10 had missing laboratory data. The demographic, clinical, and laboratory characteristics of the three groups are presented in Table 1. The mean ages of the patients in the groups were 58.7 ± 17.7, 63.8 ± 16.0, and 66.13 ± 16.0 y, respectively. The total study population comprised 119 females (44 %) and 153 males (56 %). There were significant differences between the three groups in age, coronary artery disease, white blood cell, troponin T, thrombolytic therapy, Qanadli score, RV/LV ratio, death, and combined endpoint (the need for NPPV, IPPV, and/or hemodynamic support) (*p* < 0.05). The Geneva and Wells scores were similar in the three groups. Seventy-two patients (26.5 %) were administered thrombolytics due to a diagnosis of high-risk PE (tissue plasminogen activator in nine patients, and streptokinase in sixty). Of the 359 initially included patients, thirty-six (13.2 %) died within one month of diagnosis. Of those, ten (27.7 %) were administered a thrombolytic drug, and two died due to major bleeding as a complication of thrombolytic application. Thirty deaths occurred in the hospital, and six following discharge. A significantly higher number of patients in Group 3 were treated with thrombolytic therapy compared to the other groups (Table 2).

**Table 2:** Electrocardiographic findings of the three groups on admission

Variables	Group 1 (n = 87)	Group 2 (n = 93)	Group 3 (n = 92)	*P*
Tp-e tangent (ms)	96.5 + 6.6	95.7 + 7.7	110.0 + 10.0	*0.001*
Corrected Tp-e (ms)	101.4 + 8.2	123.6 + 5.7	151.8 + 14.0	*0.001*
cQT interval (msn)	85.2 ± 9.2	94.6 ± 9.8	109.2 ± 11.4	*0.001*
Tp-e/QT	0.22 ± 0.04	0.24 ± 0.05	0.29 ± 0.07	*0.001*
QRS duration (msn)	14.9 ± 1.6	15.6 ± 1.6	16.6 ± 1.7	*0.001*
Neg T score (n)	0.9 + 1.5	2.2 + 2.0	3.8 + 2.2	*0.001*

The echocardiographic data were compared between the groups, with no difference determined in ejection fraction and pulmonary artery systolic pressure (*p* = 0.64, *p* = 0.45, respectively). There was a significant difference between the groups in RV dilatation and RV/LV ratio (*p* = 0.04); this statistically significant difference was significantly higher in Group 3 than in the other groups (Table 1).

The electrocardiographic findings of the three groups upon admission were compared, and no differences were determined in the ECG findings of left axis deviation, ST-segment depression, low voltage, findings of RV hypertrophy, or heart rate (Table 1). The median value of the cTpe was 113 ms. There was a significant difference between the groups in Tpe tangent (ms) (p = 0.001), corrected Tpe (ms) (p = 0.001), cQT interval (msn) (p = 0.001), Tpe/QT, QRS duration (msn) (p = 0.001), and negative T score (n) (p = 0.001). These differences were significantly higher in Group 3 compared to the other groups (Table 1).

**Fig. 3:**
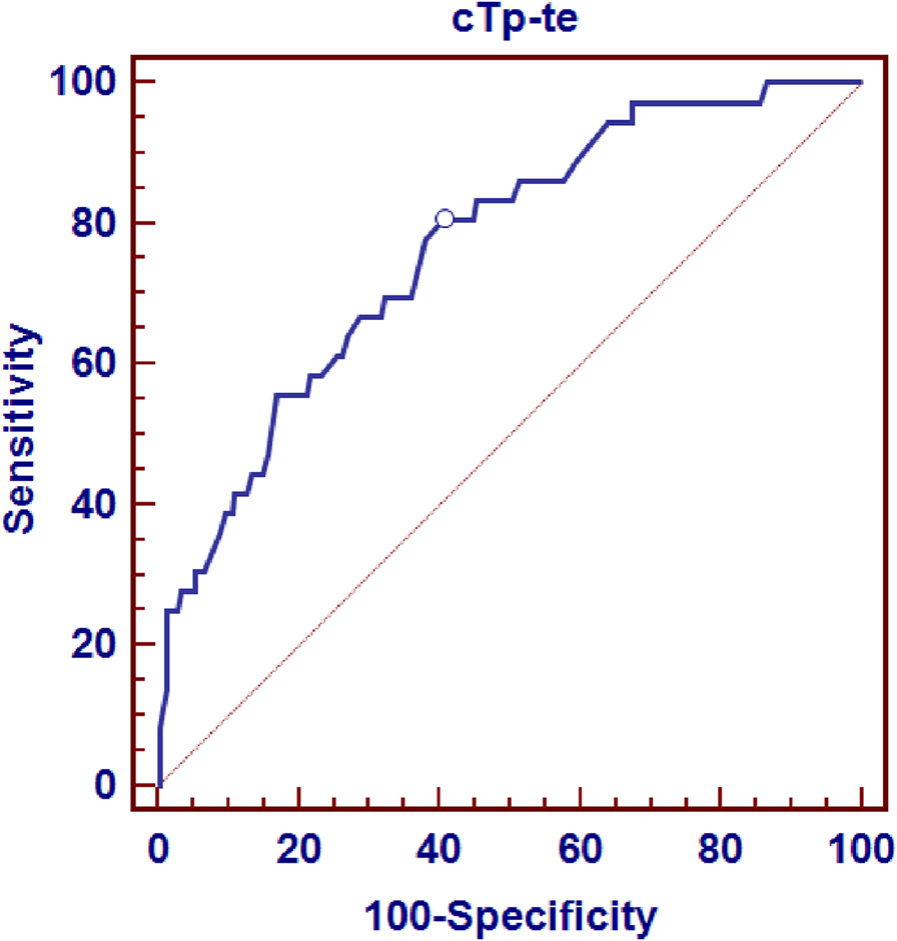
Optimal cTp-e cutoff value for 30-day mortality was determined as 126 ms with ROC analysis. cTp-e cutoff value of 126 ms had sensivity, specificity, negative predictive value and positive predictive value of 80,56 %, 59,32 %, 95,2 % and 23,2 %, respectively

**Fig. 4:**
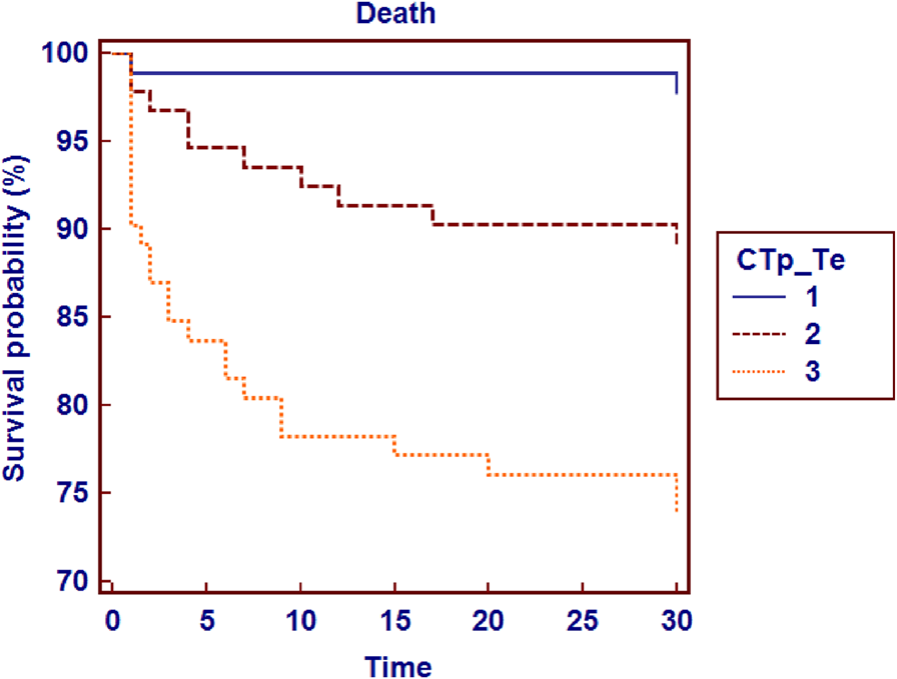
Patients with cTp-e > 126 ms had significantly lower 30-day survival rate in Kaplan- Meier curve (Fig. 4). We stratified the whole study population into cTp-e tertiles and suvival curve of the three tertile was significantly lower compared with other tertiles

In terms of adverse 30-day clinical events, there were significant differences between the groups in combined endpoint and death. Death and combined endpoint (the need for NPPV, IPPV, and/or hemodynamic support) were significantly higher in Group 3 compared to the other groups (Table 1). The optimal cTpe cut-off value for 30-day mortality was determined at 126 ms with receiver operating characteristic analysis. The cTpe cut-off value of 126 ms had sensitivity of 80.56 %, specificity of 59.32 %, negative predictive value of 95.2 %, and positive predictive value of 23.2 % (Fig. 3). Patients with cTpe > 126 ms had a significantly lower 30-day survival rate on the Kaplan-Meier curve. When the whole study population was stratified into cTpe tertiles, the survival curve of the third tertile was significantly lower than that of the other tertiles (Fig. 4).

The univariate logistic regression analyses for the predictors of all-cause mortality in the study group are presented in Table 3. The logistic regression analyses showed significant effects in the following parameters on 30-day death rates: age, ejection fraction, shock index, troponin T, white blood cell , thrombolytic therapy, and cTpe. When cTpe > 126 ms was added into the same regression model (while the independent predictors remained unchanged), the hazard ratio (HR) for cTpe significantly increased (HR: 12.9 [3.05–54.65, CI 95 %] p = 0.001). In the latter model, other independent predictors were age (HR: 1.03 [1.00–1.05, CI 95 %] p = 0. 007), shock index (HR: 25.40 [6.51–99.13, CI 95 %] p = 0.0001), troponin T (HR: 1.08 [1.01–1.15, CI 95 %] p = 0.01), white blood cell (HR: 1.11 [1.06–1.17, CI 95 %] p = 0.007), thrombolytic therapy (HR: 2.42 [1.25–4.68, CI 95 %] p = 0.001), and EF (HR: 0.94 [0.90–0.98, CI 95 %] p = 0. 005) (Table 3).

**Table 3:** Regression analyses for the predictors of all- cause mortality in the study population

Variables	Regression analysis	
	OR (95 % CI)	*p*
Age	1,03 (1,00-1,05)	*0,007*
EF	0,94 (0,90-0,98)	*0,005*
SHOCK index	25,40 (6,51- 99,13)	*0,0001*
ThrombolyticTherapy n (%)	2,42 (1,25- 4,68)	*0,008*
WBC	1,11 (1,06-1,17)	*0,0001*
Troponin T	1,08 (1,01- 1,15)	*0,01*
cTp-e	12,9 (3,05- 54,65 )	*0,001*
RVLV ratio	0,82 (0,12- 5,32)	0,83
Gender	0,82 (0,41- 1,60)	0,56
DM	1,76 (0,85-3,65)	0,12
RV dilatation (%)	1,90 (0,52-6,92)	0,32
CAD	1,58 (0,74- 3,37)	0,23

In linear relationships, the cTpe interval showed a positive correlation with heart rate, white blood cell, troponin T, RV/LV ratio, and negative T score (Table 4).

**Table 4:** Linear association between Tp-e, c Tp-e values and other variables

Variables	T p-e tangent (ms)		cT p-e tangent (ms)	
	*R*	*p*	*r*	*p*
Systolic Bp (mmHg)	−0,48	0,45	−0,92	0,15
Diastolic Bp (mmHg)	−0,41	0,52	−0,41	0,52
Heart Rate (bpm)	0,05	0,43	0,19	*0,003*
O2 saturation(%)	0,18	0,85	−0,46	0,62
SHOCK index	0,5	0,17	0,01	0,5
RVLV ratio	0,44	0,0001	0,42	*0,0001*
WBC	0,17	0,005	0,23	*0,0001*
TroponinT	0,19	0,03	0,21	*0,01*
EF	−0,10	0,22	−0,09	0,23
Neg T score	0,60	0,0001	0,55	*0,0001*

## Discussion

The present study demonstrated the prognostic value of Tpe upon hospital admission in patients with acute PE. To the best of our knowledge, little is known about the use of Tpe for identifying risk in patients with acute PE. A standard 12-lead ECG is especially useful in the differentiation of acute PE from acute coronary syndrome. Although the S1Q3T3 pattern is specific to acute PE, it is a frequently encountered pattern, which renders its diagnostic value debatable [22]. Right bundle branch block, P pulmonale, supraventricular and ventricular arrhythmias, reduced QRS amplitude, ST-segment depression, and abnormalities of cardiac repolarization (such as T-wave abnormalities) are also commonly encountered [23–25]. Vanni et al. reported that the commonly seen RV strain pattern (Right Branch Bundle Block, T negativity in precordial derivations, S1Q3T3) could predict short-term prognosis independent of normal blood pressure and echocardiograph abnormalities [26].

The most extensive study on this phenomenon was conducted by Kosuge et al., who found a relationship between the number of negative T waves seen in 12-lead ECG and the severity of acute PE, confirmed via computed tomography angiography and echocardiography. They also showed that the number of negative T waves during the course of hospitalization was the most significant predictor of death, and inotropic and mechanical cardiovascular support were secondary to hemodynamic instability [9]. Our study’s results were consistent with these works in terms of negative T scores. In another study, of 42 patients, Ding et al. showed an increase in QT and cQT dispersion calculated in the first 24 h in acute PE patients. Together with RV dysfunction, the authors reported increased cQT dispersion to be an indicator of poor prognosis [27]. Ryu et al. examined various ECG patterns seen in acute PE patients. They demonstrated that reverse T waves in V1–4 derivations and sinus tachycardia could be used in risk stratification [8].

The Tpe interval has been proposed as a more representative measure of the repolarization, because it is less dependent on heart rate, autonomic modulations, and QRS duration than the QT and QTp intervals [28]. Increased Tpe interval might be a useful index to predict ventricular tachyarrhythmias and cardiovascular mortality [29]. Previous studies have shown that prolongation of the Tpe interval is associated with increased mortality in patients with Brugada syndrome, long QT syndrome, atrioventricular nodal reentrant tachycardia, hypertrophic cardiomyopathy, and ST-segment elevation myocardial infarction [30, 31]. Our study concludes that there is a direct association between prolonged Tpe interval and increased fatal events in Group 3 patients when compared to the other two groups. To answer the question of what the optimal prolonged Tpe value is, we can answer that in healthy individuals, the mean Tpe interval is approximately 86 milliseconds in the left chest leads, similar to what others have previously reported [32].

Tpe values >100 ms during acute myocardial infarction [20] and > 113 ms in the setting of acquired bradyarrhythmias [18] have been reported previously in the literature as being “high risk”. Tatlisu et al. conducted a study in which they found that a cTpe interval of more than 114 ms was considered risky [33]. We found that patients in Group 3 had a prolonged Tpe interval, with a value of 110.0 ± 10.0 ms (versus 96.5 ± 6.6 ms in Group 1).

In the current study, the patients with prolonged Tpe/cTpe intervals had higher clinical scores and worse white blood cell values and echocardiography results. In addition, the rates of 30-day death and combined endpoints in Group 3 were higher in these patients than in the other groups. Regression analysis showed that the cTpe interval obtained in the first 24 h in acute PE patients could predict in-hospital mortality and combined endpoints. In addition, more patients with long Tpe intervals were older and female, which may show that patient groups with this profile are at greater risk. Panikkath et al. recently showed that the Tpe/QT ratio is a more accurate measurement of ventricular repolarization than QT, cQT, and Tpe durations, and is independent of heart rate [34]. In the current study, the cQT and Tpe/QT ratios of patients with prolonged cTpe intervals were high, which was consistent with the durations found in Panikkath et al.’s study. Increased cTpe intervals were associated with higher likelihood of in-hospital death and combined clinical endpoints. In addition, a Kaplan–Meier analysis showed that patients with cTpe that was longer than 126 ms more frequently reached in-hospital mortality and combined clinical endpoints.

## Conclusion

In conclusion, prolonged cTpe shows potential as a prognostic tool in early risk stratification in patients with acute PE. Future prospective studies and randomized controlled trials are warranted to validate these findings and to establish standardized cut-off values for cTpe.

### Study limitations

The primary limitations of this study are that it was retrospective, observational, and single-center, and that it examined a limited number of patients in a specific period (as such, a longer follow-up period could not be applied). It may have been useful to apply arrhythmia analysis to these patients, with Holter monitor follow-up. Another limitation may be that qualitative and/or quantitative measurement could not be applied to the RV.

## Abbreviations

cTpe: Corrected peak and end of the T wave
ECG: Electrocardiogram
HR: Hazard ratio
LV: Left-ventricular
PE: pulmonary embolism
RV: Right ventricle
Tpe: The peak and end of the T wave
